# Levels of Salivary Enzymes of *Apolygus Lucorum* (Hemiptera: Miridae), From 1^st^ Instar Nymph to Adult, and Their Potential Relation to Bug Feeding

**DOI:** 10.1371/journal.pone.0168848

**Published:** 2016-12-21

**Authors:** Xiumei Tan, Xiuping Xu, Yong Gao, Qinmin Yang, Yunsheng Zhu, Jiqing Wang, Fanghao Wan, Hongxu Zhou

**Affiliations:** 1 College of Agronomy and Plant Protection, Key Lab of Integrated Crop Pest Management of Shandong Province, Qingdao Agricultural University, Qingdao, China; 2 General Station of Plant Protection of Shandong Province, Jinan, China; 3 Institute of Plant Protection, Qingdao Academy of Agricultural Sciences, Qingdao, China; 4 State Key Laboratory for Biology of Plant Diseases and Insect Pests, Institute of Plant Protection, Chinese Academy of Agricultural Sciences, Beijing, China; Natural Resources Canada, CANADA

## Abstract

In recent years, *Apolygus lucorum* has caused increasing damage to cotton and fruit trees in China. The salivary enzymes secreted by *A*. *lucorum* when sucking on host plants induce a series of biochemical reactions in plants, and the pre-oral digestion benefits the bug feeding. In this study, the food intake of *A*. *lucorum* from 1^st^ instar nymphs to adults was measured, and the corresponding salivary activity of pectinase, amylase, cellulase, protease, polyphenol oxidase and peroxidase was determined. Daily food intake varied with developmental stage, peaking in 3^rd^ and 4^th^ instar nymphs. Pectinase, amylase, cellulase and protease were detected in both nymphal and adult saliva of *A*. *lucorum*, while neither polyphenol oxidase nor peroxidase was detected. Protease activity varied with food intake peaking at the 3^rd^-4^th^ instar, and then slightly decreasing at the 5^th^ instar. Levels of pectinase, amylase and cellulase increased significantly with the daily feeding level until the 3^rd^ instar, corresponding with increasing damage to host plants. The activity of both cellulase and protease had a significant linear relationship with the average daily food intake. The increasing activity of enzymes in saliva explain stage-specific impacts of *A*. *lucorum* on the host plants, and suggest that optimal management of *A*. *lucorum* would be confined to its control threshold prior to the peak of daily feeding in the 3^rd^ instar.

## Introduction

*Apolygus lucorum* (Meyer-Dur) (Heteroptera: Miridae), a significant pest of several key agricultural crops and fruit trees, is the most widely distributed species of mirid in China [1]. Since the late 1990s, owing to the large-scale adoption of transgenic insect-resistant cotton and the corresponding adjustment of the agricultural sector (i.e., reduction in use of pesticides in the crop), *A*. *lucorum* has replaced lepidopterous larvae such as *Helicoverpa armigera* as the primary pest in Chinese cotton fields. In addition, as an increasing acreage of fruit trees provide more host plants and suitable overwintering sites, *A*. *lucorum* has begun to gradually migrate to a wider range of crops, including jujube, peach, cherry, grape and other fruit trees, damaging buds, leaves and fruits, and causing substantial economic losses [2–4]. As a multi-host pest with good dispersal ability, *A*. *lucorum* is difficult to control [5]. During feeding, this piercing and sucking bug injects saliva into host plants to break down host tissues [6]. This is mediated by enzymes in the saliva.

Since insect saliva is injected into plants, its components may be an important means by which particular insects affect their host plants besides the direct damage caused by feeding, since enzymes and various organic compounds in the saliva of phytophagous insects can induce a series of biochemical reactions in plants [7]. When feeding, *A*. *lucorum* nymphs’ and adults’ stylet pierces into plant buds, foliar tips, young leaves, inflorescences, flower buds and young fruits, causing a variety of damage, including holes in growing tissue and deformity or complete loss of reproductive parts. In response, damaged plants often manifest increased defenses [8–10], which may be induced by the oral secretions of the insects [11]. While plants are often subject to insect herbivores, and any feeding will produce a certain degree of stress response, insects with piercing-sucking mouthparts can have a special impact on host plants, for saliva enters plant tissues deeply as opposed to just being placed on tissues eaten by a chewing insect [12–13]. Mirids feed on mesophyll using the ends of mandible and maxillae to destroy host plant cells while simultaneously excreting saliva into the wound through the stylet [14–15].

Injected saliva from species of bugs contains various digestive enzymes, whose composition varies among bug species, families, or trophic levels. For instance, the level of α-amylase activity is lower in the saliva of the predatory bug *Geocoris punctipes* than in the saliva of the phytophagous bugs *Lygus hesperus* and *Lygus lineolaris* [16]. Meanwhile, evidence of a trypsin-like enzyme, a chymotrypsin-like enzyme, and pectinase were found in the salivary glands of another predatory species, *Deraeocoris nigritulus* (Uhler) [17]. Contents of saliva may vary with the life stage of the species. For example, salivary amylase activity in the herbivorous bug *Eurygaster integriceps* increased with instar, with the least activity in the 1^st^ instar and the most in the 3^rd^ instar nymphs, with no significant difference between 3^rd^, 4^th^ and 5^th^ instar nymphs and adults [18–19].

Enzymes in insect saliva can also help degrade plant chemical defenses. For example, Urbanska et al [20] found that polyphenol oxidase enzymes in *Schizaphis graminum* saliva oxidize phenolic compounds, potentially degrading the toxicity of these substances. Peroxidase, another enzyme in saliva is believed to have a similar effect [21–22]. Compounds such as dimboa, gramine, and cinnamic acid are oxidized only by peroxidase [23]. Such effects on plant defense compounds potentially benefit herbivorous insects.

Measuring enzymes found in insect saliva has been achieved through the use of an artificial feeding system that allows for collection of saliva. Miles [24] succeeded in using nutrient solution placed in between parafilms to trap saliva from the aphid *Schizaphis graminum*, and Miles and Harrewijn [25] found that aphids feeding on sucrose presented in this manner secreted saliva with the same enzyme composition as aphids feeding on plants. Chen et al [26] also successfully fed another aphid, *Sitobion avenae*, with this parafilm-based artificial feeding method. For mirids, previous studies have examined the digestive enzymes of mirid pests as a whole, taken from the whole body, and the enzymes in saliva have not been reported. Based on the research of *A*. *lucorum* food intake at different stages, enzymes were extracted in this study from *A*. *lucorum* saliva by placing nutrient solutions between parafilm layers. The study’s goal was to evaluate the levels of these enzymes in saliva of different bug life stages and correlate these levels with stage-specific food intake. Such measures should help clarify the relative importance of feeding at different bug life stages relative to plant damage so as to control the bug at an appropriate period.

## Materials and Methods

### Ethic statement

The research complies with all laws of the country (China) and our collection of samples got the permission from the owners of the local jujube orchards in Zhanhua, Shandong Province, China.

### Experimental insects and determination of instars

Jujube (*Zizyphus jujube* Mill) twigs, about 3–4 cm, containing *A*. *lucorum* eggs were collected from a jujube orchard in Zhanhua, Shandong Province and soaked in water for 5 hours, after which 50–80 twigs were placed into one glass rearing jar (upper diameter 15 cm, bottom diameter 18 cm and height 20 cm) with a gauze cover. Following the methods of Lu et al [27],the jars were incubated at 25 ± 1°C with a 16:8 h L:D photoperiod and 70±5% relative humidity. As first instar nymphs emerged, they were transferred into another similar jar where they were offered green bean pods (*Phaseolus vulgaris* L.) for food. Each such rearing jar was initiated with 30–40 newly emerged nymphs and provided with 5–6 green bean pods, each about 10 cm long. To avoid water stress, beans were replaced with fresh material every other day.

Nymphs were categorized to instar based mainly on the size and color of wing buds. First instars lack wing buds completely and the thorax and abdomen are of equal width (in contrast to all older instars, which have wing buds and abdomens that are wider than the thorax). Second through fifth instar were recognized by the size of the wing buds, which reach progressively further back, meeting the thorax (second instar), the first abdominal segment (third instar), the third abdominal segment (fourth instar), and the fifth abdominal segment (fifth instar), respectively. In addition, the color of the wing buds darkens in the later instars, being darker than the body in the 4^th^ instar and having black tips in the 5^th^ instar. The samples in this study were collected on the first day within each nymphal instar for assaying enzymatic activity.

### Measurement of food consumption by instar

The food consumption of *A*. *lucorum* in various instars was measured using a gravimetric method [28–30]. Thirty healthy nymphs hatched simultaneously were selected and placed in a similar glass rearing jar and provided with fresh green bean pods each day at 25 ± 1°C with a 16:8 h L:D photoperiod and 70±5% relative humidity. Green bean pods were weighed with an electronic balance (precision of 0.0001 g) before and after being consumed. Moreover, the change in weight of the green bean pods due to evaporation was taken into consideration by measuring pod weights of blank controls at the same time points. Data were then corrected for dehydration during the experiment using the following formula.

Corrected food consumption was estimated as W–[L + (aW+ bL) /2], where W stands for the weight of green bean pods before consumption, L for the weight of green bean pods after consumption, a = % loss due to dehydration, estimated as (the weight of green bean pods before experiment in control group—the weight of green bean pods after experiment in control group) / the weight of green bean pods before experiment in control group, and b (the weight of green bean pods before experiment in control group—the weight of green bean pods after experiment in control group) / the weight of green bean pods after experiment in control group. Any *A*. *lucorum* nymphs that died during the experiment were replaced with fresh individuals of the same instar. The whole experiment was replicated five times.

### Collection of *A*. *lucorum* saliva

Using a nutrient solution placed between two sheets of parafilm, saliva of *A*. *lucorum* was collected as follows. Three bugs, starved for 6 h and at the same instar, were put into a transparent plastic tube open at both ends (3.5 cm diameter, 5 cm high). The upper end was covered with two layers of parafilm with 200 μL of nutrient solution sandwiched between them (20% sugar, 100 mmol/L L-serine, 100 mmol/L methionine, 100 mmol/L aspartic acid, 20 min high pressure steam sterilization, cooled and kept in the refrigerator at 4°C), and the other end covered with gauze to ensure ventilation. The tubes were held at 25 ± 0.5°C, 65 ± 1% RH, and a 16:8 h L:D photoperiod. After allowing the bugs to feed on the solution for six hours, the remaining liquid between the layers of parafilm (injected saliva and unconsumed nutrient solution) was collected. This liquid was then centrifuged for 25 min in a high-speed (15000 g/min), refrigerated (4°C) centrifuge, and the supernatant, called “enzyme-containing extract”, was collected and stored at -20°C. The whole experiment was replicated five times.

### Measurement of enzyme concentrations in saliva extracts

Protease activity was determined with the folin-phenol method [31],which defines one unit of enzyme activity as the amount of enzyme required to degrade 1 μg tyrosine from a 0.5% casein solution per minute at 40°C.

Pectolytic enzyme activity was determined using a spectrophotometer [32–34], defining one unit of enzyme activity as the amount of enzyme required to degrade 1 μg galacturonic acid from a 5 g/L pectin solution per minute at 50°C.

Amylase enzyme activity was measured using the 3,5-Dinitrosalicylic acid method [31], defining one unit of enzyme activity as the amount of enzyme required to degrade 1 μg of reducing sugars from a 1% starch solution per minute at 25°C.

Cellulase activity was detected using a spectrophotometer [35–36], defining one unit of enzyme activity as the amount of enzyme required for the degradation of 1 μg of reducing sugars from a 0.625% solution of sodium carboxymethyl cellulose per minute at 50°C and pH 4.6.

Polyphenol oxidase and peroxidase activity was detected using a spectrophotometer [37], defining one unit of enzyme activity as the change in OD (optical density) values of substrates to a value of 0.01 per milliliter of enzyme solution per minute at 37°C (for polyphenol oxidase) and 30°C (for peroxidase). Catechol was taken as substrate for the determination of polyphenol oxidase activity, and guaiacol for peroxidase activity.

Salivary enzyme secreted into nutrient solution by *A*. *lucorum* from 1^st^ instar nymph to adult was assayed using the above method. The nutrient solution was run in parallel for all enzyme assays in the same way.

### Statistical analysis

Data were processed with Excel software and analyzed with SPSS20.0 by one-way analysis of variance (ANOVA) followed by LSD multiple range test when significant differences were found at P = 0.05.

## Results

### Food consumption by different instars

Both total and daily within-stage food intake of *A*. *lucorum* bugs differed significantly among instars (Total: df = 5, F = 51.58, P = 0.000; Daily: df = 5, F = 28.514, P = 0.000) (Table 1). Total food intake corresponded to the average daily food intake before the 4^th^ instar, both increasing with larval instars. Daily food intake peaked at the 4^th^ instar and declined thereafter. However, because of the longer period of older stages, total intake of food per stage continued to increase, albeit at a slower rate, through the adult stage.

**Table 1:** The daily and total (within life stage) food consumption (as mg) of *A*. *lucorum* when fed green bean pods at 25 ± 1°C, with a 16:8 h L:D photoperiod and 70 ± 5% relative humidity.

Instars	length of instar stage(d)	Total stage-specific food consumption (mg)	PercentageOf total feeding (as %)	Daily, within life stage food consumption (mg/d)
**1st instar**	**1**	7.29 ± 0.16 d	3.16	7.29 ± 0.16 c
**2nd instar**	**2**	32.62 ± 2.44 c	14.12	16.31 ± 1.22 b
**3rd instar**	**2**	43.37 ± 1.40 b	18.77	21.69 ± 0.70 a
**4th instar**	**3**	43.68 ± 1.91 b	18.91	21.84 ± 0.96 a
**5th instar**	**3**	49.82 ± 5.22 a	21.57	16.61 ± 1.74 b
**adult**	**3**	54.22 ± 0.43 a	23.47	18.07 ± 0.14 b

### Digestive enzyme levels in saliva of *A*. *lucorum* from different life stages

**Fig 1:**
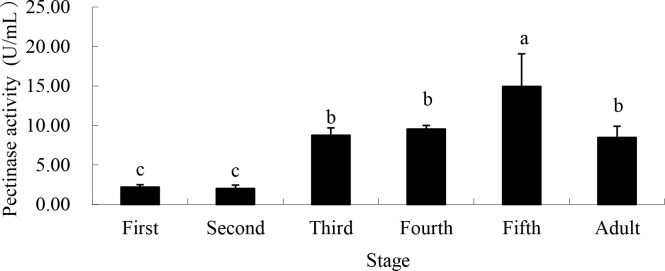
Pectinase activity from saliva of different life stages of *A*. *lucorum* when fed artificial sugar solutions between layers of parafilm. Stages with different letters are significantly different at *P* = 0.05.

Pectinase activity was lowest in 1^st^ (2.21 U/mL) and 2^nd^ (2.03 U/mL) instars, peaked in the 5^th^ instar (14.94 U/mL), and decreased in adulthood (8.48 U/mL) (Fig 1).

**Fig 2:**
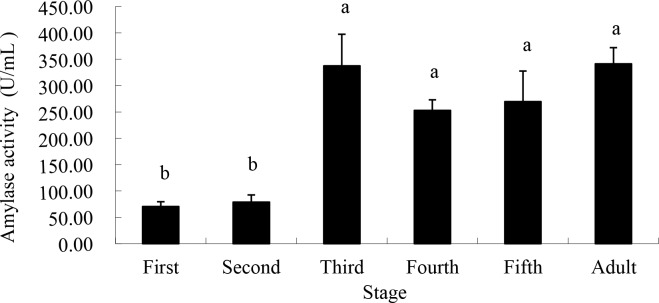
Amylase activity from saliva of different life stages of *A*. *lucorum* when fed artificial sugar solutions between layers of parafilm. Stages with different letters are significantly different at *P* = 0.05.

Amylase activity was also lowest in the 1^st^ (70.60 U/mL) and 2^nd^ (78.94 U/mL) instars, then rose in the 3^rd^ instar (337.30 U/mL) and remained at statistically identical levels thereafter, through the adult stage (Fig 2).

**Fig 3:**
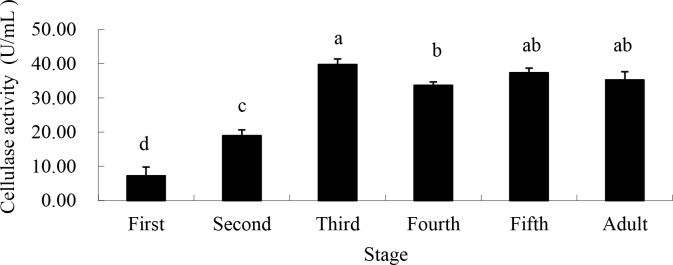
Cellulase activity from saliva of different life stages of *A*. *lucorum* when fed artificial sugar solutions between layers of parafilm. Stages with different letters are significantly different at *P* = 0.05.

Cellulase activity showed a pattern identical to that of amylase, increasing from low levels in the 1^st^ instar (7.24 U/mL), reaching relatively high levels in the 3^rd^ instar (39.76 U/mL) and remaining high thereafter, through the adult stage (Fig 3).

**Fig 4:**
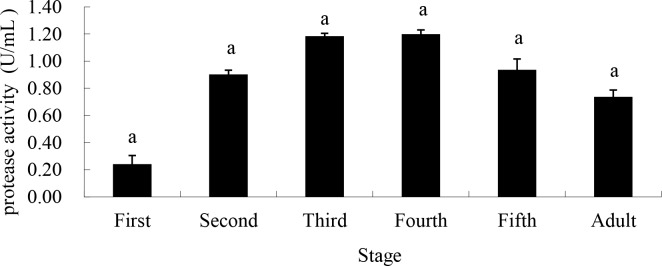
Proteinase activity from saliva of different life stages of *A*. *lucorum* when fed artificial sugar solutions between layers of parafilm. Stages with different letters are significantly different at *P* = 0.05.

Proteinase activity sample mean values were lowest in the 1^st^ instar (0.24U/mL), reached its highest in the 4^th^ instar (1.20U/mL) and then decreased in the 5^th^ (0.94U/mL) instar and adulthood (0.74U/mL). However, none of these differences were statistically significant despite large differences in means (Fig 4).

Saliva in *A*. *lucorum* bugs, tested against relevant substrates for polyphenol oxidase or peroxidase activity, showed no activity.

### Correlation of digestive enzyme levels in samples to life stage food intake

The activity of four saliva enzymes and daily food consumption at different instars were described mathematically in Table 2. The activity of pectinase and amylase showed no significant exponential functional relations to daily food intake, while cellulase and proteinase activity showed a significant linear relationship to daily food intake.

**Table 2:** Correlation of digestive enzyme levels in samples to life stage food intake

Salivary enzymes	Equation	*R*^2^	*F*	*P*
***Y***_***1***_	*Y*_*1*_ = 1.059*e*^0.102*x*^	0.426	2.966	0.160>0.05
***Y***_***2***_	*Y*_*2*_ = 31.883*e*^0.104*x*^	0.588	5.705	0.075>0.05
***Y***_***3***_	*Y*_*3*_ = 2.083*x*-6.631	0.749	11.906	0.026< 0.05
***Y***_***4***_	*Y*_*4*_ = 0.063*x*-0.210	0.913	41.886	0.003<0.05

*Y*_*1*_, *Y*_*2*_, *Y*_*3*_ and *Y*_*4*_ stand for the activity of pectinase, amylase, cellulase and proteinase respectively, and *x* stands for daily food consumption.

## Discussion

For most of their life, insects consume foods to fuel their growth and life activities. Based on substantial data on the food intake of insects with chewing-lapping mouthparts, Chen and Fan [38] found that the logarithm value of their food intake showed a linear growth in accordance with instar and also suggested that this rule may apply to piercing-sucking insects. However, research on the food intake of piercing-sucking insects has rarely been reported. Our study on *A*. *lucorum* found both total and daily food consumption increased significantly before the 4^th^ instar, implying increasing damage to plants.

Physiological structure and characterization of salivary gland complexes (SGC) of *Eurygaster integriceps* with its piercing–sucking mode of feeding showed advantages of this feeding strategy in pre-oral digestion, and the activities of salivary enzymes were increased in response to feeding [39]. Boyd etc. studied the stylet morphology and the digestive enzymes from the salivary glands and anterior midgut of *Deraeocoris nebulosus* and *Deraeocoris nigritulus* [6,17].

Various digestive enzymes have been found in the saliva of different insects, aiding in the digestion of food. The first saliva enzyme to be isolated in insects was pectinase, which enables insects to digest pectin, discovered in *Myzus persicae* (Sulzer) saliva and later in *Lygus hesperus* [40–42]. Cellulase has also been found in aphid saliva [41–42]. Various oligosaccharides depolymerases have been found in piercing-sucking insect saliva, such as exo polygalacturonase and 1,3–glucosidase found in *Schizaphis graminum* saliva, which were assumed to digest oligosaccharides in foods [41]. Amylase, which converts starch into maltose, has been found in Heteroptera (true bugs, including mirids) saliva [42–44]. A trypsin-like protease in *Lygus saliva* was discovered, which could decompose the cell wall of host plants, helpful for in vitro digestion of foods [45–47].

In our study, pectinase, amylase, cellulase and protease were all found in *A*. *lucorum* saliva enzyme, and the levels vary with bug feeding levels, illustrating the digestive functions determined in previous research. As was shown, the activity of pectinase increased significantly from the 3^rd^ instar and peaked at the 5^th^ instar, accelerating the breakdown of the pectin found in the plant cell wall, which aids the penetration of *A*. *lucorum*’s stylet into host plant tissues. The activity of amylase, cellulase and protease peaked at the 3^rd^ instar and maintained at a relatively high level until adulthood, potentially accelerating the decomposition of cellulose in the cell wall and starch in plants.

No polyphenol oxidase and peroxidase were detected in *A*. *lucorum* saliva in this study, possibly because either quite low amounts of these two enzymes make it hard to determine their activity, or because the two enzymes were not there. More research is needed to improve saliva extraction to determine whether or not these two enzymes are to be found in *A*. *lucorum* saliva at all.

Different piercing-sucking insects had different salivary components to adapt to varied hosts. Feeding on different host plants, their salivary components will change, which is an important factor in insect and plant evolution [48].The salivary components of insects with piercing-sucking mouthparts also change at different life stages, adapting to their physiological characteristics. The saliva of 1^st^ instar *Eurygaster integriceps* nymphs, which do not feed, contains very low levels of amylase, which gradually increase with age [49]. *Mayetiola destructor* (Say), on the other hand, secretes many kinds of proteins at an early developmental stage, and nymphs can accurately determine whether or not to damage plants in response to plant resistance elicited by the secretion of protease [50]. We found that pectinase, amylase and cellulase activity in *A*. *lucorum* saliva all increased with age, and were significantly higher in 3^rd^ instars than in 1^st^ instars. This change in the activity of salivary enzymes at different life stages indicated an adaptation to the requirements of plant feeding and life development.

Feeding is usually thought to intensify at late nymphal stages and adulthood. This, however, was not what we found. Daily food intake of *A*. *lucorum* in the immature stages increased constantly up to the 3^rd^ and 4^th^ instars, which showed no significant difference between the two. Daily intake was significantly lower at the 5^th^ instar than the 4^th^, and then increased slightly at adulthood. Mehrabadi et al found that activities of salivary enzymes were increased in response to feeding in comparison to starved insects, and held their production was induced by the feeding pulse [39]. Our study showed that pectinase, amylase, cellulase and protease activities in saliva were highest in 3^rd^ instar nymphs, and cellulase and proteinase had a certain relationship with daily food consumption. A significant linear relationship existed between cellulase and protease activity and food consumption. The activity level of these four salivary enzymes is directly related to the damage to host plants from insect feeding. This being so, cellulase and protease would seem to have a greater impact on the damage caused by *A*. *lucorum*.

Thus, the components and activity of salivary enzyme in *A*. *lucorum* are interconnected with its damage to host plants in a complicated way, and the damage of *A*. *lucorum* to plants is the comprehensive outcome of its saliva enzyme. The optimal management of *A*. *lucorum* should be confined to a control threshold prior to the peak of daily feeding level, which our results suggest peaks, along with salivary enzyme activity, at 3–4 instars. This reinforces the conventional control phase in field management at an early nymph stage, usually before the 3^rd^ instar. These findings will help inform the outbreak mechanism of *A*. *lucorum* by elucidating the levels of salivary enzymes related to the damage on host plants within each nymphal instar and adult, suggesting the optimal timing on the bug management.

## Supporting Information

S1 Table Enzyme activity of pectinase, amylase, cellulase and protease secreted by the salivary glands of *Apolygus lucorum*. (DOCX)
