# Supplementary material for: Levels of Salivary Enzymes of Apolygus Lucorum (Hemiptera: Miridae), From 1st Instar Nymph to Adult, and Their Potential Relation to Bug Feeding
Source: PLoS One. 2016 Dec 21;11(12):e0168848. doi: 10.1371/journal.pone.0168848 (PMC5176182; doi:10.1371/journal.pone.0168848)
Supplement: S1 Table — (DOCX) [file pone.0168848.s001.docx]

**S1 Table. Enzyme activity of pectinase, amylase, cellulase and protease secreted by the salivary glands of** ***Apolygus lucorum***

| Mirid stage | Pectinase activity (U/mL ) | Amylase activity (U/mL ) | Cellulase activity (U/mL ) | Protease activity (U/mL ) |
| --- | --- | --- | --- | --- |
| First instar | 2.21 | 70.60 | 7.24 | 0.24 |
| Second instar | 2.03 | 78.94 | 18.99 | 0.90 |
| Third instar | 8.76 | 337.30 | 39.76 | 1.18 |
| Fourth instar | 9.55 | 252.78 | 33.69 | 1.20 |
| Fifth instar | 14.94 | 269.68 | 37.38 | 0.94 |
| Adult | 8.48 | 341.12 | 35.25 | 0.74 |
